# SBAV: A Sex-Stratified Precision Framework for Predicting Sleep Apnea–Driven Neurodegeneration in Aging Women

**DOI:** 10.1093/geroni/igaf122.4257

**Published:** 2025-12-31

**Authors:** Hanan Sheikh Ibrahim

**Affiliations:** Cleveland Clinic Abu Dhabi, Abu Dhabi, Abu Dhabi, United Arab Emirates

## Abstract

Background Obstructive sleep apnea (OSA) is a known risk factor for cognitive impairment, yet remains underrecognized in aging women. Traditional tools such as STOP-BANG and apnea–hypopnea index (AHI) thresholds were validated in male cohorts, failing to account for hormonal decline, altered sleep architecture, and sex-specific neuroinflammation post-menopause. Objective To develop and present the SBAV (Sleep–Brain–Aging Vulnerability) framework—a multi-modal, sex-stratified precision model integrating genomics, electrophysiology, hormonal markers, and AI-supported analytics to improve early detection of OSA-associated cognitive risk in women. Methods A structured scoping review (2018–2024) was conducted using PubMed, Scopus, and Web of Science. Key domains included: - Genomics (APOE ε4, CRY1, CLOCK) - Proteomics (tau, amyloid-β, IL-6, BDNF) - Endocrine markers (estrogen, cortisol) - EEG sleep microarchitecture (spindle loss, REM/NREM fragmentation) - Neuroimaging features (hippocampal atrophy) Findings were synthesized into a sex-stratified dashboard linking EEG and biomarker data with cognitive profiles. Results Women with OSA demonstrate a distinct EEG phenotype: reduced spindle density, altered slow-wave activity, and delayed cortical arousal, correlated with elevated tau and memory decline. Hormonal depletion and APOE ε4 synergistically amplify neurodegenerative vulnerability under intermittent hypoxia. Standard diagnostics miss over 40% of female cases. SBAV integrates omics, EEG, and hormonal data to outperform AHI-based stratification by 35–42%, enabling earlier, sex-specific risk identification. Conclusion SBAV redefines OSA in women as a sex-dependent neurodegenerative amplifier, proposing a paradigm shift in sleep and dementia screening. By leveraging AI-assisted, multi-omic stratification, this model lays the groundwork for tailored women’s cognitive prevention in midlife.

